# Association of *ABCB1* and *FLT3* Polymorphisms with Toxicities and Survival in Asian Patients Receiving Sunitinib for Renal Cell Carcinoma

**DOI:** 10.1371/journal.pone.0134102

**Published:** 2015-08-05

**Authors:** Ying-Hsia Chu, Huihua Li, Hui Shan Tan, Valerie Koh, Johnathan Lai, Wai Min Phyo, Yukti Choudhury, Ravindran Kanesvaran, Noan Minh Chau, Chee Keong Toh, Quan Sing Ng, Puay Hoon Tan, Balram Chowbay, Min-Han Tan

**Affiliations:** 1 Institute of Bioengineering and Nanotechnology, Singapore, Republic of Singapore; 2 Health Services Research, Singapore General Hospital, Singapore, Republic of Singapore; 3 Division of Medical Oncology, National Cancer Centre Singapore, Singapore, Republic of Singapore; 4 Department of Pathology, Singapore General Hospital, Singapore, Republic of Singapore; 5 Laboratory of Clinical Pharmacology, Division Medical Sciences, National Cancer Centre Singapore, Singapore, Republic of Singapore; 6 Clinical Pharmacology Core, SingHealth, Singapore, Republic of Singapore; 7 Clinical Sciences, Duke-NUS Graduate Medical School, Singapore, Republic of Singapore; Sapporo Medical University, JAPAN

## Abstract

Sunitinib is a tyrosine kinase inhibitor used as first-line treatment for metastatic renal cell carcinoma (mRCC). Asian ethnicity has been previously associated with lower clearance and greater toxicities for sunitinib treatment, relative to Caucasian ethnicity. Research focusing on identifying corresponding biomarkers of efficacy and toxicity has been hitherto conducted in Caucasian populations, and few of the reported associations have been externally validated. Our work thus aims to investigate candidate biomarkers in Asian patients receiving sunitinib, comparing the observed genotype effects with those reported in Caucasian populations. Using data from 97 Asian mRCC patients treated with sunitinib, we correlated 7 polymorphisms in *FLT3*, *ABCB1*, *VEGFR2*, *ABCG2* and *BIM* with patient toxicities, response, and survival. We observed a stronger association of *FLT3 738T* genotype with leucopenia in our Asian dataset than that previously reported in Caucasian mRCC patients (odds ratio [OR]=8.0; *P*=0.03). We observed significant associations of *FLT3 738T* (OR=2.7), *ABCB1 1236T* (OR=0.3), *ABCB1 3435T* (OR=0.1), *ABCB1 2677T* (OR=0.4), *ABCG2 421A* (OR=0.3) alleles and *ABCB1 3435*, *1236*, *2677 TTT* haplotype (OR=0.1) on neutropenia. Primary resistance (OR=0.1, *P*=0.004) and inferior survival (progression-free: hazard ratio [HR]=5.5, *P*=0.001; overall: HR=5.0, *P*=0.005) were associated with the *ABCB1 3435*, *1236*, *2677 TTT* haplotype. In conclusion, *ABCB1* and *FLT3* polymorphisms may be helpful in predicting sunitinib toxicities, response and survival benefit in Asian mRCC patients. We have also validated the association between *FLT3 738T* and sunitinib-induced leucopenia previously reported in Caucasian populations, but have not validated other reported genetic associations.

## Introduction

Sunitinib is a tyrosine kinase inhibitor that targets vascular endothelial growth factor receptors (VEGFR1, VEGFR2 and VEGFR3), platelet-derived growth factors (PDGFRα and PDGFβ), Fms-like tyrosine kinase 3 (FLT3) and the RET protein [1–4]. It is used as a standard treatment of metastatic renal cell carcinoma (mRCC) in the first-line setting. Although sunitinib has demonstrated benefits in comparison with interferon therapy [4], clinical outcomes including best radiological response, survival and toxicities are heterogeneous, with 25% of patients achieving complete or partial response and 57% exhibiting severe adverse effects in the recent COMPARZ trial [5]. Sunitinib-associated toxicities include diarrhea, hand-foot syndrome, mucositis, hypertension, leucopenia, neutropenia and thrombocytopenia, as well as abnormalities in hepatic, renal, pancreatic and left ventricular function [4]. In the landmark phase 3 trial, toxicities led to dose interruption in 38% and dose reduction in 32% of patients [4]. Asian patients have been noted to experience higher toxicities from sunitinib therapy. For instance, the incidences of grade 3 to 4 thrombocytopenia (37.7%), neutropenia (29.5%) and anemia (21.9%) reported in Korean patients [6] were more than double of the incidences reported in Western patients [4, 7, 8]. This might be related to a previous observation that Asian ethnicity is associated with decreased sunitinib clearance as compared to Caucasians [9].

Since the FDA approval of sunitinib in 2006, genetic biomarkers have received intense research attention as a promising measure to personalize sunitinib use by profiling individual toxicity and response predisposition. To this end, several prior studies (S1 Table) have correlated survival outcome and toxicity incidences with several single nucleotide polymorphisms (SNPs) in the genes that encode sunitinib targets (such as *VEGFR1* [10], *VEGFR2* [11, 12], *PDGFRα* [11], *FLT3* [11] and *FLT4* [13, 14]), other proteins involved in proangiogenic pathways (*VEGFA* [14–17], *FGFR2* [13] and *eNOS* [17]), hepatic xenobiotic-metabolizing enzymes (*CYP3A5* [18] and *CYP1A1* [11, 19]), hepatic enzyme modulators (*NR1/3* [13] and *NR1/2* [13]), as well as transcellular multidrug efflux pumps such as *ABCG2* [11, 20] and *ABCB1* [8, 11, 21]. However, few of these reported associations have been replicated, except for an association between *VEGFR3 3971 GG* genotype and better progression-free survival in two independent studies [13, 18]. The majority of these previous studies were conducted in North America and Europe. In comparison, little work has been done to study Asian populations, an interesting demographic to study given the high incidences of grade 3 to 4 toxicities present in Asian populations [6].

We identified three candidate polymorphisms, *1236C/T*, *3435C/T* and *2677G/T* of *ABCB1* for their demonstrated effect on the functionality of the multi-specificity transporter encoded [22, 23]. Sunitinib is a substrate of ABCB1 and another efflux transporter encoded by *ABCG2*; brain accumulation of sunitinib has been observed to increase significantly in *ABCB1* knockout and *ABCB1/ABCG2* double knockout mice, despite bioavailability after oral dosing remaining similar to that of wild type mice [24]. Recently, *ABCB1 1236C/T* and *2677G/T* were found to be associated with the clearance of sunitinib in a study involving 114 cancer patients in the Netherlands [25]. Currently, the three *ABCB1* polymorphisms have been associated with hand-foot syndrome and survival in sunitinib receivers in exploratory studies [11, 13, 19] in Europe. Given these findings and known interethnic allele frequency variations (for instance, the *ABCB1 1236 T* allele was found in 71.9% and 41% of a Chinese [26] and German population [27] respectively), we were interested in investigating the correlation of *ABCB1* polymorphisms with sunitinib treatment outcomes in Asian patients.

Recently, a 2,903-base-pair deletion polymorphism in intron 2 of the *BIM* gene was found to be associated with unfavorable outcomes upon treatment with multiple tyrosine kinase inhibitors (TKIs). For example, inferior imatinib response in chronic myelogenous leukemia and shorter progression-free survival in EGFR—mutated non—small-cell lung cancer treated with gefitinib or erlotinib was observed in an Asian population [28]. The likely underlying mechanism is alternate splicing leading to loss of the pro-apoptotic BCL2-homology domain 3 (BH3) [28]. The involvement of *BIM* in sunitinib activity has been suggested by several prior animal and *in vitro* studies—Naik *et al*. demonstrated that destruction of tumor vasculature by VEGF-blocking antibodies was *BIM*-dependent [29] and Yang *et al*. noted that there was upregulation of *BIM* along with other proapoptotic genes in human medulloblastoma cell lines treated with sunitinib [30]. The investigation of the association of *BIM* deletion with outcomes in sunitinib-receiving patients is therefore a subject of interest to us.

This study aimed to evaluate genetic polymorphisms to investigate their association with sunitinib toxicities and survival benefits in Asian renal cancer patients. We have selected candidate polymorphisms based on previously reported effects in Caucasian patients in order to compare the genotype effects seen in each ethnicity. We anticipated that the increased prevalence of high-grade toxicities in Asians would yield increased statistical power in determining relevant genetic markers.

## Materials and Methods

### Patients and treatment

A total of 97 mRCC patients who received sunitinib between 2006 and 2014 at the National Cancer Centre Singapore (NCCS) were included in this retrospective study. The study was approved by the Institutional Review Board (Singapore Health Services) and written informed consent was obtained from each patient. Sample size estimation is detailed in S5 Table. The majority of patients (79/97) received sunitinib at a starting dose of 37.5 mg daily over 4 consecutive weeks followed by a 2 week break. This attenuation and deviation from the drug label-recommended dosage of 50mg daily was established as routine at NCCS after severe to life-threatening toxicities were frequently noted when sunitinib was initiated at 50mg daily. Efficacy outcomes as determined through a national retrospective analysis have been comparable [31]. 12 patients in this study received a starting dose of 50mg daily. 6 patients received a starting dose of 25mg daily due to advanced age or an aversion to the expected toxicities.

### Follow-up and data collection

Sunitinib toxicities and best radiological response were evaluated based on CTCAE version 3.0 [32] and RECIST criteria version 1.1 [33]. Laboratory assessments of serum creatinine, total bilirubin, albumin, aspartate transaminase (AST), alanine transaminase (ALT), hemoglobin, leucocytes and platelets and clinical examinations for hand-foot syndrome and diarrhea were conducted at baseline (before starting sunitinib) and at two time points in each cycle: after 4 weeks of daily sunitinib and after 2 weeks of sunitinib-free rest (before starting the next cycle). Patient characteristics including age, gender, self-reported ethnicity, body weight and height and Eastern Cooperative Oncology Group (ECOG) performance status were also collected. Memorial Sloan-Kettering Cancer Center (MSKCC) prognostic score [34] was calculated for each patient with the available data. All collected data was de-identified by a third party before being used in statistical analysis. The follow-up period ended at the end of April, 2014.

### Toxicity definitions

The toxicities assessed include leucopenia, neutropenia, thrombocytopenia, hepatotoxicity, diarrhea and hand-foot syndrome. Blood cell counts from the electronic medical system and physician notes from the first sunitinib cycle were assessed for leucopenia (<3000/μL), neutropenia (<2000/μL), thrombocytopenia (<150000/μL), hand-foot syndrome (documented physical examination findings) and diarrhea (documented patient complaints). Hepatotoxicity was defined as elevation of AST (>33IU/L) or ALT (>36IU/L) above a normal baseline (AST≤33IU/L and ALT≤36IU/L) during the first two cycles.

### Survival endpoint definition

Progression-free survival (PFS) was defined as the time from the date of sunitinib initiation to the date of sunitinib termination when sunitinib was terminated due to radiological or clinical evidence of progressive disease (PD), severe toxicities or death, with termination due to PD and death due to PD as events. Dose reduction did not count as an endpoint for PFS. Overall survival (OS) was defined as the time from the date of sunitinib initiation to the date of death or to the date of the last follow-up for censored cases.

### Genotyping

We genotyped 6 SNPs in 4 genes, including *FLT3 738 T/C*, *VEGFR2 1191C/T*, *ABCG2 421C/A*, *ABCB1 3435C/T*, *ABCB1 1236T/C*, *ABCB1 2677G/TA*, as well as an intron 2 deletion polymorphism of *BIM* [28]. The SNPs were selected based on minor allele frequency higher than 0.1 in Han Chinese, previously reported associations with sunitinib toxicities (S1 Table) and presumed function in sunitinib pharmacokinetics or pharmacodynamics. Primers for genotyping the SNPs and the *BIM* deletion are provided in S6 Table [15, 35, 36].

Germline DNA was obtained from the buffy coat or from formalin-fixed tissue of benign kidney obtained from nephrectomy. The labeling on blood tubes and tissue slides were de-identified by a third party before they were used for DNA extraction. Genotyping was done by PCR amplification of the flanking region of each SNP followed by direct sequencing.

### Statistical analysis

Genotype associations with toxicity events or best radiological response were first analyzed using univariate logistic regression. Genotypes generating *P*<0.20 were further analyzed using multivariate logistic regression including patient age, gender, baseline ECOG status and starting dose as covariates. PFS and OS were estimated using the Kaplan-Meier method [37]. Univariate associations of genotypes and patient characteristics with PFS and OS were analyzed using either a two-tailed log rank test [38] or a Cox proportional hazard test depending on the property of the variable. Genotypes generating *P*<0.20 were further analyzed using a multivariate Cox regression model by including patient characteristics which had univariate *P* values of less than 0.05 as covariates and PFS or OS as the depending variable. Only patients for whom sunitinib was the first line treatment for mRCC were included in PFS and OS analyses. In all analyses, missing data were kept missing except for baseline ECOG status, which was replaced with the median value. With an exploratory purpose, multiple testing correction was not done.

## Results

### Patient characteristics and genotype frequencies

The demographic and baseline clinical characteristics of the 97 patients included in this study are listed in Table 1. The polymorphism frequencies of the 6 SNPs and the *BIM* deletion are listed in Table 2. Hardy-Weinberg equilibrium held for all 6 SNPs and the BIM deletion (***P***>0.05) [39]. After verifying pairwise linkage disequilibrium for ***ABCB1 3435C/T*, *ABCB1 1236C/T*** and ***ABCB1 2677G/TA*** using a Chi-square test (***P***< 0.05 in each pair) and phasing with PLINK [40], haplotype ***TTT*** was found to be the most common haplotype. It was found in 51 patients, among whom 8 were homozygous carriers. A complete list of haplotypes and their frequencies is provided in S2 Table.

**Table 1:** Patient demographics and baseline characteristics (n = 97).

Characteristic	No.		%
**Median age when initiating sunitinib, years**		58	
Range		18–79	
**Gender**			
Male	75		77.3
Female	22		22.7
**Ethnicity (self-reported)**			
Chinese	86		88.7
Malay	7		7.2
Indian	4		4.1
**Baseline ECOG performance status**			
0	27		27.8
1	51		52.6
2	13		13.4
3	6		6.2
**Median body surface area, m** ^**2**^		1.63	
Range		1.18–1.92	
**Line of therapy**			
First line	81		83.5
Second or third line	16		16.5
**Starting sunitinib dose, mg daily**			
25	6		6.2
37.5	79		81.4
50	12		12.4
**Baseline chemistry and hematology (n < 97 due to missing data)**			
Median aspartate transaminase, U/L (n = 93)		23	
Range		11–70	
Median alanine transaminase, U/L (n = 93)		20	
Range		8–135	
Median creatinine, μM (n = 96)		104	
Range		33–649	
Median hemoglobin, g/dL (n = 96)		11.5	
Range		5.9–15.8	
Median leukocyte, K/μL (n = 96)		7.3	
Range	1.4–24.1		
Median thrombocyte, K/μL (n = 96)		280	
Range	117–799		

**Table 2:** Polymorphisms genotyped and allele frequencies.

				Genotype distribution			
Polymorphism	rs number	Variation	n^a^	wt/wt	wt/var	var/var	VAF^e^
*VEGFR2 1191 C/T*	rs2305948	V297I	94	64	30	0	0.160 (*T*)
*FLT3 738 T/C*	rs1933437	M227T	95	47	43	5	0.279 (*C*)
*ABCB1 1236 T/C*	rs1128503	G412G	93	35	49	9	0.360 (*C*)
*ABCB1 2677 G/TA*	rs2032582	A893S/T	96	25	44^b^	27^c^	0.375 (*T*); 0.135 (*A*)
*ABCB1 3435 C/T*	rs1045642	I1145I	96	34	50	12	0.385 (*T*)
*ABCG2 421 C/A*	rs2231142	Q141K	95	50	38	7	0.274 (*A*)
*BIM* i2del ^d^	-	i2del ^d^	45	33	12	0	0.133

^a^ Patients successfully genotyped.

^b^ Includes 34 *GT* and 10 *AG* individuals.

^c^ Includes 2 *AA*, 12 *AT* and 13 *TT* individuals.

^d^ A 2,903-bp deletion polymorphism in intron 2 of *BIM* previously associated with resistance to tyrosine kinase inhibitors [28]. As we were unable to genotype formalin-fixed tissues with the current method, only 45 patients were typed.

^e^ Variant allele frequencies.

### Correlation of genotypes to toxicities

Univariate and multivariate logistic regression analyses for associations between genetic markers and clinical outcomes are listed in Table 3 (non-significant results are provided in S3 Table). It is noteworthy that the *FLT3 738 TT* genotype was associated with an 8.0-fold increase in the risk of leucopenia (*P* = 0.03) and a 2.7-fold increase in the risk of neutropenia (*P* = 0.04). The *ABCB1 1236 T* allele, *ABCB1 3435 T* allele, *ABCB1 2677 T* allele, *ABCB1 3435*, *1236*, *2677 TTT* haplotype and the *ABCG2 421 A* allele were correlated with a 3-fold (P = 0.03), 10-fold (P = 0.01), 3-fold (P = 0.04), 10-fold (P = 0.03) and 3-fold (P = 0.03) decrease in the risk of neutropenia respectively. The *ABCB1 1236 T* and *ABCB1 3435 T* alleles were correlated with a 25-fold (P = 0.0005) and 3-fold (P = 0.02) decrease in the risk of diarrhea respectively. No genotypes were correlated with thrombocytopenia, hepatotoxicity or hand-foot syndrome. The *VEGFR2 1191C/T* genotype and *BIM* deletion were not associated with the toxicity endpoints.

**Table 3:** Factors associated with toxicities of sunitinib.

			Univariate		Multivariate^a^	
Group		Prevalence^b^	OR (95% CI)	*P*	OR (95% CI)	*P*
**Leucopenia (n = 85)**						
Age		11/85	1.0 (0.9, 1.0)	0.44		
Gender	Male vs.	6/65	1			
	Female	5/20	3.3 (0.9, 12.4)	0.08		
Baseline ECOG	0	1/25	1			
	1	9/44	6.2 (1.1, 117.6)	0.09		
	2	1/12	2.2 (0.1, 58.7)	0.59		
	3	0/4	NR	0.99		
Starting dose (mg)	≤37.5	0/4	1			
	37.5	10/70	NR	0.99		
	50	1/11	NR	0.99		
*FLT3 738 T/C*	*CC*+*CT*	2/42	1		1	
	*TT*	8/41	4.9 (1.1, 33.6)	0.06	8.0 (1.3, 51.0)	0.03
*BIM* i2del^d^	Wild type	4/29	1		1	
	Deletion	1/10	0.7 (0.0, 5.5)	0.76	NR	0.39
**Neutropenia (n = 88)**						
Age		40/88	1.0 (1.0, 1.1)	0.24		
Gender	Male	27/68	1			
	Female	13/20	2.8 (1.0, 8.4)	0.05		
Baseline ECOG	0	13/25	1			
	1	22/46	0.9 (0.3, 2.3)	0.74		
	2	5/12	0.7 (0.2, 2.6)	0.56		
	3	0/5	NR	0.99		
Starting dose (mg)	≤37.5	1/5	1			
	37.5	32/72	3.2 (0.5, 64.3)	0.31		
	50	7/11	7.0 (0.7, 165.7)	0.13		
*FLT3 738 T/C*	*CC*+*CT*	15/45	1		1	
	*TT*	23/41	2.6 (1.1, 6.2)	0.04	2.7 (1.1, 7.2)	0.04
*ABCG2 421 C/A*	*CC*+*AC*	23/42	1		1	
	*AA*	16/44	0.5 (0.2, 1.1)	0.09	0.3 (0.1, 0.9)	0.03
*ABCB1 1236 T/C*	*CC+CT*	27/52	1		1	
	*TT*	11/32	0.5 (0.2, 1.2)	0.12	0.3 (0.1, 0.9)	0.03
*ABCB1 2677G/TA*	Other	20/35	1		1	
	*TT*+*AT*+*GT*	20/53	0.5 (0.2, 1.1)	0.08	0.4 (0.1, 0.9)	0.04
*ABCB1 3435 C/T*	*CC+CT*.	38/75	1		1	
	*TT*	1/12	0.1 (0.0, 0.5)	0.02	0.1 (0.0, 0.4)	0.01
*ABCB1* haplotype^c^	Other	38/79	1		1	
	*TTT/TTT*	1/8	0.2 (0.0, 0.9)	0.09	0.1 (0.0, 0.5)	0.03
*BIM* i2del^d^	Wild type	13/27	1		1	
	Deletion	6/11	1.3 (0.3, 5.5)	0.72	NR	0.93
**Diarrhea (n = 95)**						
Age		20/95	1.0 (1.0, 1.0)	0.62		
Gender	Male	15/74	1			
	Female	5/21	1.2 (0.4, 3.7)	0.73		
Baseline ECOG	0	6/27	1			
	1	9/50	0.8 (0.2, 2.6)	0.66		
	2	4/12	1.8 (0.4, 7.9)	0.47		
	3	1/6	0.7 (0.0, 5.6)	0.76		
Starting dose (mg)	≤37.5	2/5	1			
	37.5	15/78	0.4 (0.1, 2.9)	0.28		
	50	3/12	0.5 (0.1, 5.2)	0.54		
*ABCB1 3435 T/C*	*CC*	11/34	1		1	
	*TT+CT*	9/60	0.4 (0.1, 1.0)	0.05	0.3 (0.1, 0.8)	0.02
*ABCB1 1236 T/C*	*CC*	7/9	1		1	
	*TT+CT*	13/82	0.1 (0.0, 0.3)	0.0006	0.04 (0.0, 0.2)	0.0005
*BIM* i2del^d^	Wild type	7/33	1		1	
	Deletion	5/12	2.7 (0.6, 11.2)	0.18	3.1 (0.6, 16.5)	0.17

Abbreviations: OR, ratio of the odds that the event occurs; CI, confidence interval; NR, not reached; PR, partial response; SD, stable disease.

^a^ Including age, gender, starting dose and baseline ECOG status as covariates.

^b^ Number of cases affected by toxicity/ total number of cases in the group.

^c^ *ABCB1 3435C/T*, *1236C/T*, *2677G/TA* haplotype.

^d^ A 2,903-bp deletion polymorphism in intron 2 of *BIM* previously associated with resistance to tyrosine kinase inhibitors [28]. As we were unable to genotype formalin-fixed tissues with the current method, only 45 patients were typed.

### Correlation of genotypes with best radiological response and patient survival

**Fig 1:**
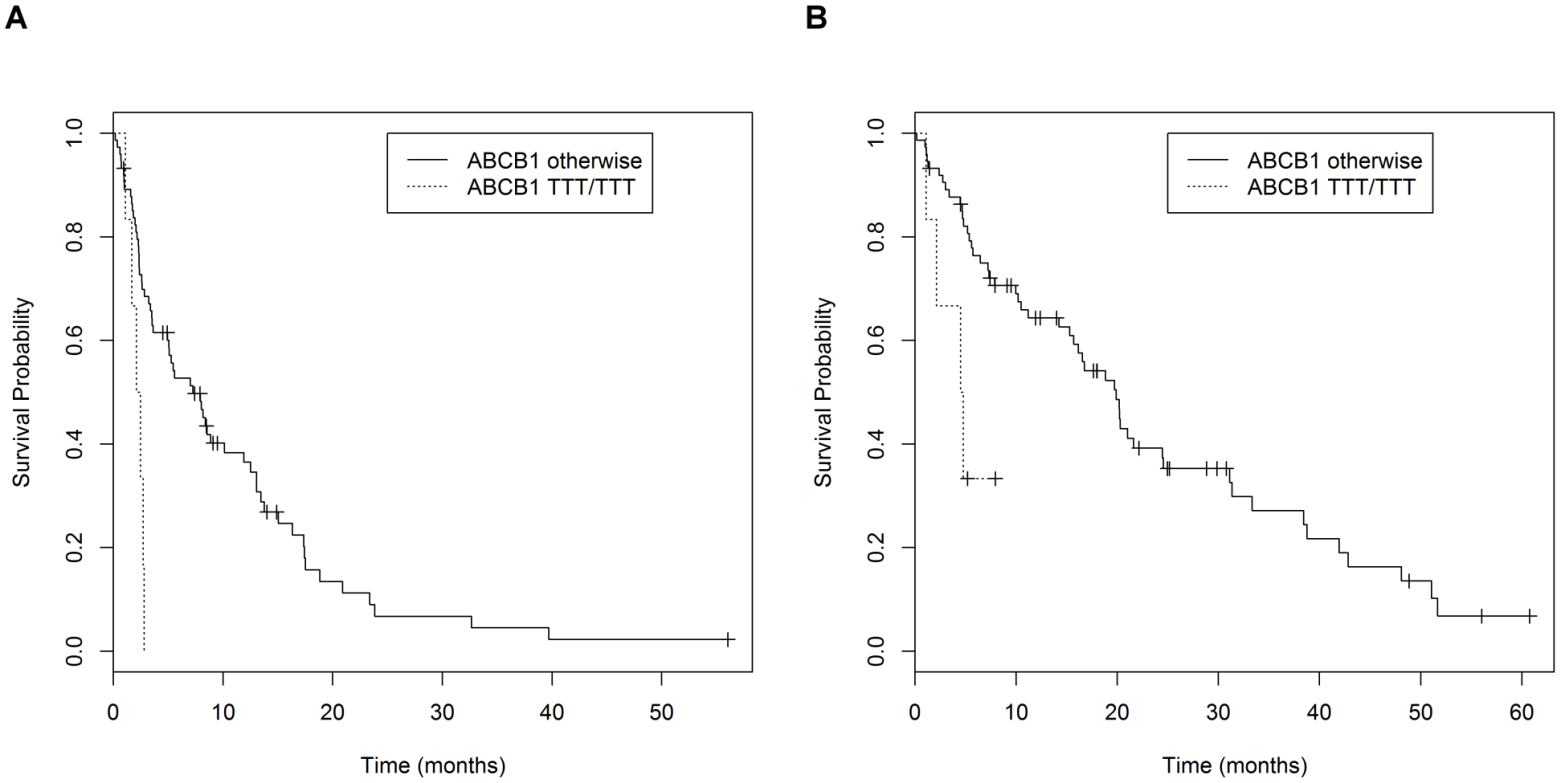
Survival curves. (A) Patients grouped according to the *ABCB1 3435C/T*, *1236C/T*, *2677G/T* haplotype; median PFS was 2.4 months for homozygous carriers of the *TTT* haplotype and 8.4 months for other cases (*P* = 0.001). (B) Patients grouped according to the *ABCB1 3435C/T*, *1236C/T*, *2677G/TA* haplotype; median OS was 4.6 months for homozygous carriers of the *TTT* haplotype and 19.6 months for other cases (*P* = 0.005).

Primary sunitinib resistance, defined as the condition in which progressive disease is the best radiological response observed, was more common in carriers of the *ABCB1 3435 TT* genotype (*P* = 0.02), *ABCB1 2677 TT* genotype (*P* = 0.01) and the *ABCB1 3435*, *1236*, *2677 TTT* haplotype (*P* = 0.004) (Table 4). Median PFS of the 81 patients who received sunitinib as the first-line therapy was 8.1 months and median OS was 19.5 months. As shown in Table 5 (non-significant results are provided in S4 Table), after including starting dose as a covariate based on univariate *P*<0.05, the *ABCB1 3435*, *1236*, *2677 TTT* haplotype was correlated with inferior PFS (*P* = 0.001) and OS (*P* = 0.005) (survival curves are provided in Fig 1).

**Table 4:** Factors associated with the clinical benefit of sunitinib (best response being PR or SD) (n = 90).

			Univariate		Multivariate^a^	
Group		Prevalence^b^	OR (95% CI)	*P*	OR (95% CI)	*P*
Age		59/90	1.0 (0.9, 1.0)	0.38		
Gender	Male	46/71	1			
	Female	13/19	1.2 (0.4, 3.7)	0.77		
Baseline ECOG	0	20/25	1			
	1	31/49	0.4 (0.1, 1.3)	0.15		
	2	5/11	0.2 (0.0, 0.9)	0.05		
	3	3/5	0.4 (0.1, 3.4)	0.35		
Starting dose (mg)	≤37.5	1/4	1			
	37.5	49/75	5.7 (0.7, 117.5)	0.14		
	50	9/11	13.5 (1.1, 378.2)	0.06		
*VEGFR2 1191 C/T*	*CC*	42/60	1		1	
	*CT*	14/27	0.5 (0.2, 1.2)	0.11	0.5 (0.2, 1.3)	0.16
*FLT3 738 T/C*	*CC*+*CT*	29/46	1		1	
	*TT*	29/43	1.2 (0.5, 2.9)	0.66	1.1 (0.4, 2.8)	0.84
*ABCG2 421 C/A*	*CC*+*AC*	25/39	1		1	
	*AA*	32/49	1.1 (0.4, 2.5)	0.91	0.8 (0.3, 2.1)	0.62
*ABCB1 1236 T/C*	*CC+CT*	37/54	1		1	
	*TT*	19/33	0.6 (0.3, 1.5)	0.30	0.5 (0.2, 1.3)	0.13
*ABCB1 2677 G/TA*	Other	55/79	1		1	
	*TT*	4/11	0.3 (0.1, 0.9)	0.04	0.1 (0.0, 0.6)	0.01
*ABCB1 3435 C/T*	*CC+CT*.	54/78	1		1	
	*TT*	5/12	0.3 (0.1, 1.1)	0.07	0.2 (0.0, 0.7)	0.02
*ABCB1* haplotype^c^	Other	57/82	1		1	
	*TTT/TTT*	2/8	0.2 (0.0, 0.7)	0.02	0.1 (0.0, 0.3)	0.004
*BIM* i2del^d^	Wild type	25/33	1		1	
	Deletion	8/10	1.3 (0.3, 9.6)	0.78	1.0 (0.2, 8.4)	0.97

Abbreviations: OR, ratio of the odds that the event occurs; CI, confidence interval; NR, not reached; PR, partial response; SD, stable disease.

^a^ Including age, gender, starting dose and baseline ECOG status as covariates.

^b^ Number of cases with PR or SD as the best response observed / total number of cases in the group.

^c^ *ABCB1 3435C/T*, *1236C/T*, *2677G/TA* haplotype.

^d^ A 2,903-bp deletion polymorphism in intron 2 of *BIM* previously associated with resistance to tyrosine kinase inhibitors [28]. As we were unable to genotype formalin-fixed tissues with the current method, only 45 patients were typed.

**Table 5:** Survival analyses in mRCC patients receiving sunitinib as first-line treatment (n = 81).

			Median	Univariate		Multivariate^a^	
Factor		No.	(months)	HR (95% CI)	*P*	HR (95% CI)	*P*
**Progression-free survival**							
Age		81	8.1	1.0 (1.0, 1.0)	0.56		
Gender	Female	20	10.0	1	0.69		
	Male	61	8.1	1.1 (0.6, 2.1)			
Baseline ECOG	0	21	16.1	1	0.08		
	1	43	6.9	2.2 (1.1, 4.4)			
	2	11	3.3	2.8 (1.1, 7.0)			
	3	6	12.9	2.8 (0.8, 10.3)			
Starting dose (mg)	≤37.5	5	1.8	1	0.01		
	37.5	71	8.3	0.2 (0.1, 0.8)			
	50	5	17.3	0.1 (0.0, 0.6)			
MSKCC	Good	7	12.6	1	0.14		
	Intermediate	33	10.0	1.3 (0.5, 3.6)			
	Poor	23	5.5	2.3 (0.8, 6.4)			
*ABCB1 1236 T/C*	*CC+CT*	51	11.7	1	0.09	1	0.09
	*TT*	28	3.6	1.7 (0.9, 3.0)		1.7 (0.9, 3.2)	
*ABCB1 2677 G/TA*	Other	71	8.4	1	0.09	1	0.44
	*TT*	10	2.7	2.3 (0.9, 6.0)		1.5 (0.5, 4.7)	
*ABCB1 3435 C/T*	*CC+CT*.	70	8.4	1	0.19	1	0.19
	*TT*	10	2.7	1.7 (0.8, 3.9)		1.7 (0.8, 3.9)	
Haplotype ^b^	Other	74	8.4	1	0.0006	1	0.001
	*TTT/TTT*	6	2.4	4.9 (1.8, 13.6)		5.5 (2.0, 15.4)	
*BIM* i2del ^c^	Wild type	30	12.3	1	0.28	1	0.47
	Deletion	10	7.9	1.6 (0.7, 4.0)		1.4 (0.6, 3.7)	
**Overall survival**							
Age		81	19.5	1.0 (1.0, 1.0)	0.56		
Gender	Female	20	19.9	1	0.87		
	Male	61	16.3	1.1 (0.6, 2.0)			
Baseline ECOG	0	21	32.9	1	0.06		
	1	43	19.6	1.9 (0.9, 3.9)			
	2	11	5.7	3.0 (1.3, 7.1)			
	3	6	15.7	2.7 (0.9, 8.0)			
Starting dose (mg)	≤37.5	5	4.6	1	<0.0001		
	37.5	71	19.5	0.2 (0.1, 0.4)			
	50	5	47.4	0.1 (0.0, 0.3)			
MSKCC	Good	7	41.4	1	0.10		
	Intermediate	33	19.6	2.2 (0.8, 6.5)			
	Poor	23	14	3.1 (1.0, 9.1)			
*ABCB1 1236 T/C*	*CC+CT*	51	20	1	0.09	1	0.07
	*TT*	28	10.4	1.7 (0.9, 2.9)		1.7 (1.0, 3.1)	
*ABCB1 2677 G/TA*	Other	71	19.6	1	0.01	1	0.12
	*TT*	10	5.9	2.9 (1.3, 6.7)		2.0 (0.8, 5.0)	
*ABCB1 3435 C/T*	*CC+CT*.	70	19.5	1	0.25	1	0.21
	*TT*	10	7.2	1.6 (0.7, 3.6)		1.7 (0.7, 3.8)	
Haplotype^b^	Other	74	19.6	1	0.008	1	0.005
	*TTT/TTT*	6	4.6	3.9 (1.3, 11.7)		5.0 (1.6, 15.2)	
*BIM* i2del^c^	Wild type	30	24.1	1	0.78	1	0.49
	Deletion	10	16.3	0.8 (0.2, 3.0)		0.6 (0.2, 2.3)	

Abbreviations: HR, hazard ratio; CI, confidence interval.

^a^ Including starting dose as covariate.

^b^ *ABCB1 3435C/T*, *1236C/T*, *2677G/TA* haplotype.

^c^ A deletion polymorphism in intron 2 of *BIM* [28].

## Discussion

We observed that the *FLT3 738 TT* genotype predisposed to sunitinib-related leucopenia, an association which had previously been previously reported by van Erp *et al*. in Caucasian patients [11]. The effect size we observed i.e. an 8.0-fold increase in risk was greater than the 2.4-fold increase previously reported [11]. This may be related to interethnic differences in allele frequencies of other potentially leucopenia-predisposing genotypes such as the *CYP1A1 2455A/G* (the *G* allele is present in 3% of Caucasians and 26% of Chinese based on NCBI data) noted by van Erp *et al*. [11] but not included in this study. Houk *et al*. also correlated Asian ethnicity with a 13% decrease in sunitinib clearance and 15% increase in peak serum sunitinib concentration and area under curve compared to a control group that was composed of >85% Caucasians [9]. The increased drug exposure, for which interethnic differences in polymorphism frequencies could potentially play a role, may have an influence on the effect sizes of genotype-toxicity associations.

The *ABCB1 2677T* allele was associated with reduced neutropenia risk and inferior radiological response and the *ABCB1 1236 T* allele was associated with reduced risk of neutropenia and diarrhea. A trend was observed for the association of the *ABCB1 1236 T* allele with inferior PFS (*P* = 0.09) and OS (*P* = 0.07), which was previously reported in Caucasians [13]. This association appears to be in accordance with the findings of Diekstra *et al*., whose study correlated *ABCB1 1236 TT* and *ABCB1 2677 TT* to increased clearance of sunitinib and its active metabolite in 114 cancer patients using univariate analyses that did not include demographic covariates [25]. It is also congruent with Beuselinck *et al*.’s findings that mRCC patients who received sunitinib as first-line therapy and carried *ABCB1 1236 TT* or *ABCB1 2677 TT/TA* require fewer dose reductions due to toxicities compared to carriers of other genotypes [21]. One plausible hypothesis is that increased clearance leads to decreased drug exposure, reduced toxicity and inferior response. However, Diekstra *et al*. noted that the effect size of a single genetic polymorphism on clearance is much smaller than that of inter-individual variability and is thus inadequate to directly guide dosing [25]. Therefore, the discovery of a panel of genetic markers that collectively offers adequate predictive power and the addition of non-genetic (eg. demographic) markers into the model remain to be investigated.

Our observation that the *ABCG2 421 AA* genotype was associated with reduced risk of neutropenia (which we defined as <2000/μL being equivalent to grade 1 and above as described in CTCAE version 3.0 [32]) appears to be inconsistent with the observation of Kim *et al*. [20] that grade 3 or grade 4 neutropenia is significantly more common in carriers of this genotype. In comparison with the Korean cohort (n = 65) studied by Kim *et al*. [20], among whom 61.5% were first-line sunitinib receivers, 83.5% of our mostly Chinese cohort of patients were first-line sunitinib receivers. Furthermore, 81.4% of our patients started treatment with a reduced dose (37.5mg daily) from the standard course (50mg daily). Although further studies are required for clarification, these differences may possibly explain the discordant observations.

The limitations of this study include the retrospective nature of our data collection and the attenuated dosing regimens adopted in Singapore to reduce toxicity. Indeed, we observed lower toxicity incidences as compared to that of the recent COMPARZ trial [5]. For example, 13%, 49%, 46% and 21% of our cohort developed leucopenia, thrombocytopenia, neutropenia and diarrhea respectively. However, the survival outcome we observed (median PFS: 8.1 months; median OS: 19.5 months) is similar to that observed previously by van der Veldt *et al*. [19] (median PFS: 10.0 months; median OS: 16.3 months), whose study of a cohort of 136 mRCC patients employed the standard 50mg daily dose and calculated PFS and OS from the day of sunitinib initiation. Furthermore, we included starting dose in the multivariate analyses for each genotype correlation with toxicities, response and survival to avoid confounding effect produced by uneven dosing in the genotype models.

## Conclusion

Based on our findings, *ABCB1* and *FLT3* polymorphisms may be helpful in predicting sunitinib toxicities, response and survival benefit in Asian mRCC patients. We have validated the predisposition to leucopenia associated with *FLT3* polymorphism as has been previously reported in Caucasian populations.

## Supporting Information

S1 Table Previously reported SNPs with effect on outcomes of sunitinib treatment. (DOC)

S2 Table *ABCB1* haplotype frequencies estimated with and without assuming associations. (DOC)

S3 Table Factors with non-significant association with toxicities of sunitinib. (DOC)

S4 Table Genotypes with non-significant associations with survival in mRCC patients receiving sunitinib as first-line treatment. (DOC)

S5 Table Sample size estimation for the validation of previous associations. (DOC)

S6 Table Primers for Genotyping. (DOC)
