# Supplementary material for: Association of ABCB1 and FLT3 Polymorphisms with Toxicities and Survival in Asian Patients Receiving Sunitinib for Renal Cell Carcinoma
Source: PLoS One. 2015 Aug 5;10(8):e0134102. doi: 10.1371/journal.pone.0134102 (PMC4526634; doi:10.1371/journal.pone.0134102)
Supplement: S1 Table — (DOC) [file pone.0134102.s001.doc]

| S1 Table. Previously reported SNPs with effect on outcomes of sunitinib treatment | | | |
| --- | --- | --- | --- |
| SNP | Significantly associated outcomes | Disease and cohort size | Country^a^ |
| ***VEGFR1*** | | | |
| rs9582036 | Poorer OS with *CC* genotype. | mRCC (n=91) | France, Belgium |
| rs9554320 | Poorer PFS with *AA* genotype. | mRCC (n=91) | France, Belgium |
| ***VEGFR2*** | | | |
| *1191 C/T* | Any toxicity grade>2 increased with *T* allele. | mRCC, GIST (n=183) | Netherlands |
| *1718T>A* | Poorer PFS and OS with the *AA* genotype. | gastric, biliary (n=63) | Korea |
| ***FLT3*** | | | |
| *738 T/C* | Leucopenia increased with *T* allele. | mRCC, GIST (n=188) | Netherlands |
| ***FLT4 (VEGFR3)*** | | | |
| rs6877011  *3971 G/T* | Better PFS and OS with the *CC* genotype.  Better PFS but poorer OS with the *GG* genotype.  Better PFS with the *GG* genotype. | mRCC (n=84)  mRCC (n= 88)  mRCC (n=89) | Italy  France, Belgium  Spain |
| *1480 A/G* | Better OS with the *AA* genotype.  Better PFS with the *AA* genotype. | mRCC (n= 88)  mRCC (n=89) | France, Belgium  Spain |
| ***ABCB1*** | | | |
| *1236 T/C* | Poorer PFS and OS with the *TT* genotype.  Less dose reductions with the *TT* genotype. | mRCC (n= 88)  mRCC (n= 96) | France, Belgium  France, Belgium |
| *2677G/TA* | Less dose reductions with *TT or TA* genotypes. | mRCC (n= 96) | France, Belgium |
| haplotype^b^ | More HFS with *TTT* haplotype.  Better PFS increased with *TCG* haplotype. | mRCC, GIST (n=219)  mRCC (n= 136) | Netherlands  Netherlands |
| ***ABCG2*** | | | |
| *421 C/A* | More thrombocytopenia, neutropenia, and HFS with the *AA* genotype. | mRCC (n=65) | Korea |
| haplotype^c^ | Any toxicity grade>2 increased with *TT* haplotype. | mRCC, GIST (n=183) | Netherlands |
| ***CYP1A1*** | | | |
| *2455A/G* | More leucopenia and mucositis with *G* allele. | mRCC, GIST (n=193) | Netherlands |
| ***CYP3A5*** | | | |
| 6986G/A | Dose reduction more often with *G* allele.  Better PFS with the *A* allele. | mRCC (n=89)  mRCC (n= 136) | Spain  Netherlands |
| ***NR1/2*** | | | |
| *8055 C/T* | Poorer PFS with the *TT* genotype. | mRCC (n= 88) | France, Belgium |
| ***NR1/3*** | | | |
| *7837 T/G* | Poorer PFS and OS with the *TT* genotype. | mRCC (n= 88) | France, Belgium |
| *5719 C/T* | Better PFS with the *CC* genotype. | mRCC (n= 88) | France, Belgium |
| haplotype^d^ | Less leucopenia with *CAG* haplotype.  Poorer PFS with *CAT* haplotype. | mRCC, GIST (n=188)  mRCC (n= 136) | Netherlands  Netherlands |
| ***VEGFA*** | | | |
| rs2010963 | More hypertension with the *G* allele.  Better PFS with the *G* allele. | mRCC (n=63)  mRCC (n=84) | USA  Italy |
| *-1498 C/T* | More hypothyroidism with the *C* allele.  Poorer PFS and OS with the *TT* genotype. | GIST (n=39)  mRCC (n=84) | Poland  Italy |
| *936 C/T* | More hypothyroidism with the *T* allele. | GIST (n=39) | Poland |
| haplotype^e^ | More severe hypertension with *ACG* haplotype. | mRCC, GIST (n=255) | Netherlands |
| ***FGFR2*** | | | |
| *906C/T* | Poorer PFS with the *TT* genotype. | mRCC (n= 88) | France, Belgium |
| ***eNOS*** | | | |
| rs2070744 | Less grade 3 hypertension with *TT* genotype. | mRCC, GIST (n=255) | Netherlands |
| Abbreviations: OS, overall survival; PFS, progression-free survival; HFS, hand-foot syndrome; GIST, gastrointestinal stromal tumor.  ^a^ where the study was conducted.  ^b^ the *3435C/T, 1236C/T, 2677G/TA* haplotype*.*  ^c^ the *-15622C/T, 1143C/T* haplotype.  ^d^ the *5719C/T, 7738A/C, 7837T/G* haplotype.  ^e^ the rs699947, rs833061, rs2010963 haplotype. | | | |

**References for this table:**

(1) Beuselinck, B. *et al.* VEGFR1 single nucleotide polymorphisms associated with outcome in patients with metastatic renal cell carcinoma treated with sunitinib - a multicentric retrospective analysis. *Acta oncologica (Stockholm, Sweden)* **53**, 103-12 (2014).

(2) van Erp, N.P. *et al.* Pharmacogenetic pathway analysis for determination of sunitinib-induced toxicity. *J Clin Oncol* **27**, 4406-12 (2009).

(3) Maeng, C.H. *et al.* Effects of single nucleotide polymorphisms on treatment outcomes and toxicity in patients treated with sunitinib. *Anticancer research* **33**, 4619-26 (2013).

(4) Scartozzi, M. *et al.* VEGF and VEGFR polymorphisms affect clinical outcome in advanced renal cell carcinoma patients receiving first-line sunitinib. *British journal of cancer* **108**, 1126-32 (2013).

(5) Beuselinck, B. *et al.* Single-nucleotide polymorphisms associated with outcome in metastatic renal cell carcinoma treated with sunitinib. *British journal of cancer* **108**, 887-900 (2013).

(6) Garcia-Donas, J. *et al.* Single nucleotide polymorphism associations with response and toxic effects in patients with advanced renal-cell carcinoma treated with first-line sunitinib: a multicentre, observational, prospective study. *Lancet Oncol* **12**, 1143-50 (2011).

(7) Beuselinck, B. *et al.* Efflux pump ABCB1 single nucleotide polymorphisms and dose reductions in patients with metastatic renal cell carcinoma treated with sunitinib. *Acta oncologica (Stockholm, Sweden)*, 1-10 (2014).

(8) van der Veldt, A.A. *et al.* Genetic polymorphisms associated with a prolonged progression-free survival in patients with metastatic renal cell cancer treated with sunitinib. *Clin Cancer Res* **17**, 620-9 (2011).

(9) Kim, H.R. *et al.* Pharmacogenetic determinants associated with sunitinib-induced toxicity and ethnic difference in Korean metastatic renal cell carcinoma patients. *Cancer chemotherapy and pharmacology* **72**, 825-35 (2013).

(10) Kim, J.J. *et al.* Association of VEGF and VEGFR2 single nucleotide polymorphisms with hypertension and clinical outcome in metastatic clear cell renal cell carcinoma patients treated with sunitinib. *Cancer* **118**, 1946-54 (2012).

(11) Rutkowski, P. *et al.* The outcome and predictive factors of sunitinib therapy in advanced gastrointestinal stromal tumors (GIST) after imatinib failure - one institution study. *BMC Cancer* **12**, 107 (2012).

(12) Eechoute, K. *et al.* Polymorphisms in endothelial nitric oxide synthase (eNOS) and vascular endothelial growth factor (VEGF) predict sunitinib-induced hypertension. *Clinical pharmacology and therapeutics* **92**, 503-10 (2012).
