# Supplementary material for: Association of ABCB1 and FLT3 Polymorphisms with Toxicities and Survival in Asian Patients Receiving Sunitinib for Renal Cell Carcinoma
Source: PLoS One. 2015 Aug 5;10(8):e0134102. doi: 10.1371/journal.pone.0134102 (PMC4526634; doi:10.1371/journal.pone.0134102)
Supplement: S2 Table — (DOC) [file pone.0134102.s002.doc]

| S2 Table. *ABCB1* haplotype frequencies estimated with and without assuming associations (n=92) | | | | |
| --- | --- | --- | --- | --- |
| 3435 C/T | 1236 T/C | 2677 G/TA | Without association | With association |
| C | C | A | 0.028 | 0.096 |
| C | C | G | 0.114 | 0.222 |
| C | C | T | 0.083 | 0.000 |
| C | T | A | 0.049 | 0.029 |
| C | T | G | 0.199 | 0.214 |
| C | T | T | 0.146 | 0.059 |
| T | C | A | 0.017 | 0.000 |
| T | C | G | 0.070 | 0.037 |
| T | C | T | 0.051 | 0.008 |
| T | T | A | 0.030 | 0.000 |
| T | T | G | 0.122 | 0.032 |
| T | T | T | 0.089 | 0.303 |
