# Supplementary material for: Association of ABCB1 and FLT3 Polymorphisms with Toxicities and Survival in Asian Patients Receiving Sunitinib for Renal Cell Carcinoma
Source: PLoS One. 2015 Aug 5;10(8):e0134102. doi: 10.1371/journal.pone.0134102 (PMC4526634; doi:10.1371/journal.pone.0134102)
Supplement: S3 Table — (DOC) [file pone.0134102.s003.doc]

| S3 Table. Factors with non-significant association with toxicities of sunitinib | | | | | | |
| --- | --- | --- | --- | --- | --- | --- |
| Factor | No. of events | No. of patients | Uncorrected | | Corrected by age, gender,  ECOG and Dose | |
|  |  |  | OR (95% CI) | P-value | OR (95% CI) | P-value |
| **Leucopenia (n=85)** | | | | | | |
| ***ABCB1* haplotype ^a^** |  |  |  |  |  |  |
| other/other | 6 | 39 | Reference |  | Reference |  |
| *TTT/TTT*+*TTT*/other | 4 | 45 | 0.54 (0.13, 2.03) | 0.3645 | 0.61 (0.14, 2.62) | 0.5060 |
| ***ABCB1* haplotype ^a^** |  |  |  |  |  |  |
| other/other+*TTT*/other | 10 | 76 | Reference |  | Reference |  |
| *TTT/TTT* | 0 | 8 | - | 0.9942 | - | 0.9970 |
| **rs1045642** |  |  |  |  |  |  |
| *CC+CT* | 10 | 72 | Reference |  | Reference |  |
| *TT* | 0 | 12 | - | 0.9929 | - | 0.9970 |
| **rs1045642** |  |  |  |  |  |  |
| *CC* | 5 | 28 | Reference |  | Reference |  |
| *TT+CT* | 5 | 56 | 0.45 (0.11, 1.77) | 0.2419 | 0.62 (0.14, 2.65) | 0.5150 |
| **rs2032582** |  |  |  |  |  |  |
| *AA+AG+GG+AT+GT* | 9 | 72 | Reference |  | Reference |  |
| *TT* | 2 | 13 | 1.27 (0.18, 5.83) | 0.7759 | 2.48 (0.30, 16.2) | 0.3500 |
| **rs2032582** |  |  |  |  |  |  |
| *AA+AG+GG* | 5 | 33 | Reference |  | Reference |  |
| *TT+AT+GT* | 6 | 52 | 0.73 (0.20, 2.74) | 0.6296 | 0.62 (0.14, 2.58) | 0.5030 |
| **rs1128503** |  |  |  |  |  |  |
| *CC+CT* | 6 | 50 | Reference |  | Reference |  |
| *TT* | 4 | 31 | 1.09 (0.26, 4.15) | 0.9044 | 1.11 (0.24, 5.11) | 0.8890 |
| **rs1128503** |  |  |  |  |  |  |
| *CC* | 1 | 8 | Reference |  | Reference |  |
| *TT+CT* | 9 | 73 | 0.98 (0.15, 19.5) | 0.9888 | 0.70 (0.06, 8.39) | 0.7770 |
| **rs2231142** |  |  |  |  |  |  |
| *CC+AC* | 6 | 41 | Reference |  | Reference |  |
| *AA* | 5 | 42 | 0.79 (0.21, 2.84) | 0.7143 | 0.51 (0.10, 2.28) | 0.3967 |
| **rs2305948** |  |  |  |  |  |  |
| *CC* | 8 | 54 | Reference |  | Reference |  |
| *CT* | 3 | 28 | 0.69 (0.14, 2.63) | 0.6069 | 0.72 (0.14, 3.06) | 0.6700 |
| **Thrombocytopenia (n=86)** | | | | | | |
| **Age** | 42 | 86 | 1.05 (1.01, 1.09) | 0.0213 |  |  |
| **Gender** |  |  |  |  |  |  |
| Male | 29 | 66 | Reference |  |  |  |
| Female | 13 | 20 | 2.37 (0.86, 7.03) | 0.1038 |  |  |
| **Baseline ECOG** |  |  |  |  |  |  |
| 0 | 15 | 25 | Reference |  |  |  |
| 1 | 21 | 45 | 0.58 (0.21, 1.56) | 0.2867 |  |  |
| 2 | 5 | 12 | 0.48 (0.11, 1.91) | 0.2986 |  |  |
| 3 | 1 | 4 | 0.22 (0.01, 2.02) | 0.2194 |  |  |
| **Starting dose** |  |  |  |  |  |  |
| <37.5mg | 3 | 4 | Reference |  |  |  |
| 37.5mg | 33 | 71 | 0.29 (0.01, 2.38) | 0.2930 |  |  |
| 50mg | 6 | 11 | 0.40 (0.02, 4.36) | 0.4822 |  |  |
| ***ABCB1* haplotype ^a^** |  |  |  |  |  |  |
| other/other | 16 | 38 | Reference |  | Reference |  |
| *TTT/TTT*+*TTT*/other | 25 | 47 | 1.56 (0.66, 3.74) | 0.3102 | 1.61 (0.61, 4.33) | 0.3402 |
| ***ABCB1* haplotype ^a^** |  |  |  |  |  |  |
| other/other+*TTT*/other | 38 | 77 | Reference |  | Reference |  |
| *TTT/TTT* | 3 | 8 | 0.62 (0.12, 2.69) | 0.5262 | 0.37 (0.06, 2.00) | 0.2500 |
| **rs1045642** |  |  |  |  |  |  |
| *CC+CT* | 37 | 73 | Reference |  | Reference |  |
| *TT* | 4 | 12 | 0.49 (0.12, 1.69) | 0.2717 | 0.39 (0.08, 1.69) | 0.2207 |
| **rs1045642** |  |  |  |  |  |  |
| *CC* | 12 | 29 | Reference |  | Reference |  |
| *TT+CT* | 29 | 56 | 1.52 (0.62, 3.83) | 0.3638 | 1.61 (0.57, 4.63) | 0.3703 |
| **rs2032582** |  |  |  |  |  |  |
| *AA+AG+GG+AT+GT* | 36 | 73 | Reference |  | Reference |  |
| *TT* | 6 | 13 | 0.88 (0.26, 2.90) | 0.8337 | 0.63 (0.15, 2.45) | 0.4981 |
| **rs2032582** |  |  |  |  |  |  |
| *AA+AG+GG* | 14 | 32 | Reference |  | Reference |  |
| *TT+AT+GT* | 28 | 54 | 1.38 (0.58, 3.37) | 0.4681 | 1.20 (0.45, 3.22) | 0.7196 |
| **rs1128503** |  |  |  |  |  |  |
| *CC+CT* | 25 | 50 | Reference |  | Reference |  |
| *TT* | 15 | 33 | 0.83 (0.34, 2.01) | 0.6852 | 0.64 (0.23, 1.72) | 0.385 |
| **rs1128503** |  |  |  |  |  |  |
| *CC* | 3 | 8 | Reference |  | Reference |  |
| *TT+CT* | 37 | 75 | 1.62 (0.37, 8.37) | 0.5273 | 0.96 (0.16, 5.93) | 0.9636 |
| **rs1933437** |  |  |  |  |  |  |
| *CC+CT* | 23 | 46 | Reference |  | Reference |  |
| *TT* | 18 | 38 | 0.9 (0.38, 2.13) | 0.8102 | 0.75 (0.27, 2.00) | 0.5627 |
| **rs2231142** |  |  |  |  |  |  |
| *CC+AC* | 18 | 39 | Reference |  | Reference |  |
| *AA* | 23 | 45 | 1.22 (0.52, 2.90) | 0.6505 | 1.18 (0.43, 3.25) | 0.7408 |
| **rs2305948** |  |  |  |  |  |  |
| *CC* | 27 | 55 | Reference |  | Reference |  |
| *CT* | 14 | 29 | 0.97 (0.39, 2.39) | 0.9434 | 0.83 (0.30, 2.33) | 0.7278 |
| **Neutropenia (n=88)** | | | | | | |
| ***ABCB1* haplotype ^a^** |  |  |  |  |  |  |
| other/other | 24 | 41 | Reference |  | Reference |  |
| *TTT/TTT*+*TTT*/other | 15 | 46 | 0.34 (0.14, 0.81) | 0.0165 | 0.27 (0.09, 0.71) | 0.0110 |
| **rs1045642** |  |  |  |  |  |  |
| *CC* | 19 | 30 | Reference |  | Reference |  |
| *TT+CT* | 20 | 57 | 0.31 (0.12, 0.77) | 0.0134 | 0.26 (0.09, 0.74) | 0.0141 |
| **rs2032582** |  |  |  |  |  |  |
| *AA+AG+GG+AT+GT* | 37 | 75 | Reference |  | Reference |  |
| *TT* | 3 | 13 | 0.31 (0.07, 1.10) | 0.0915 | 0.23 (0.04, 0.96) | 0.0573 |
| **rs1128503** |  |  |  |  |  |  |
| *CC* | 5 | 8 | Reference |  | Reference |  |
| *TT+CT* | 33 | 76 | 0.46 (0.09, 2.01) | 0.3114 | 0.21 (0.03, 1.15) | 0.0890 |
| **rs2305948** |  |  |  |  |  |  |
| *CC* | 26 | 56 | Reference |  | Reference |  |
| *CT* | 13 | 29 | 0.94 (0.38, 2.31) | 0.8883 | 0.85 (0.32, 2.25) | 0.7415 |
| **Diarrhea (n=95)** | | | | | | |
| ***ABCB1* haplotype ^a^** |  |  |  |  |  |  |
| other/other | 13 | 45 | Reference |  | Reference |  |
| *TTT/TTT*+*TTT*/other | 7 | 49 | 0.41 (0.14, 1.12) | 0.0892 | 0.35 (0.11, 1.03) | 0.065 |
| ***ABCB1* haplotype ^a^** |  |  |  |  |  |  |
| other/other+*TTT*/other | 19 | 86 | Reference |  | Reference |  |
| *TTT/TTT* | 1 | 8 | 0.5 (0.03, 3.09) | 0.5331 | 0.57 (0.03, 4.02) | 0.6270 |
| **rs1045642** |  |  |  |  |  |  |
| *CC+CT* | 18 | 82 | Reference |  | Reference |  |
| *TT* | 2 | 12 | 0.71 (0.10, 3.02) | 0.6773 | 0.71 (0.1, 3.3) | 0.6920 |
| **rs2032582** |  |  |  |  |  |  |
| *AA+AG+GG+AT+GT* | 19 | 82 | Reference |  | Reference |  |
| *TT* | 1 | 13 | 0.28 (0.01, 1.54) | 0.2307 | 0.26 (0.01, 1.61) | 0.2280 |
| **rs2032582** |  |  |  |  |  |  |
| *AA+AG+GG* | 11 | 38 | Reference |  | Reference |  |
| *TT+AT+GT* | 9 | 57 | 0.46 (0.17, 1.25) | 0.1279 | 0.39 (0.13, 1.14) | 0.0905 |
| **rs1128503** |  |  |  |  |  |  |
| *CC+CT* | 16 | 57 | Reference |  | Reference |  |
| *TT* | 4 | 34 | 0.34 (0.09, 1.04) | 0.0776 | 0.36 (0.09, 1.16) | 0.1070 |
| **rs1933437** |  |  |  |  |  |  |
| *CC+CT* | 7 | 47 | Reference |  | Reference |  |
| *TT* | 12 | 46 | 2.02 (0.73, 5.96) | 0.1854 | 2.11 (0.74, 6.47) | 0.1730 |
| **rs2231142** |  |  |  |  |  |  |
| *CC+AC* | 11 | 44 | Reference |  | Reference |  |
| *AA* | 9 | 49 | 0.67 (0.24, 1.82) | 0.4384 | 0.68 (0.24, 1.94) | 0.4730 |
| **rs2305948** |  |  |  |  |  |  |
| *CC* | 11 | 62 | Reference |  | Reference |  |
| *CT* | 9 | 30 | 1.99 (0.71, 5.52) | 0.1857 | 2.26 (0.76, 6.84) | 0.1390 |
| **Elevation of alanine transaminase from a normal baseline (n=64)** | | | | | | |
| **Age** | 14 | 64 | 1.03 (0.98, 1.09) | 0.3233 |  |  |
| **Gender** |  |  |  |  |  |  |
| Male | 9 | 45 | Reference |  |  |  |
| Female | 5 | 19 | 1.43 (0.38, 4.93) | 0.5777 |  |  |
| **Baseline ECOG** | NA | NA |  |  |  |  |
| 0 | 6 | 21 | Reference |  |  |  |
| 1 | 4 | 32 | 0.36 (0.08, 1.44) | 0.1530 |  |  |
| 2 | 3 | 9 | 1.25 (0.21, 6.60) | 0.7944 |  |  |
| 3 | 1 | 2 | 2.50 (0.09, 70.6) | 0.5398 |  |  |
| **Starting dose** |  |  |  |  |  |  |
| <37.5mg | 0 | 1 | Reference |  |  |  |
| 37.5mg | 11 | 54 | - | 0.9922 |  |  |
| 50mg | 3 | 9 | - | 0.9918 |  |  |
| ***ABCB1* haplotype ^a^** |  |  |  |  |  |  |
| other/other | 7 | 31 | Reference |  | Reference |  |
| *TTT/TTT*+*TTT*/other | 7 | 33 | 0.92 (0.28, 3.07) | 0.8947 | 0.58 (0.14, 2.23) | 0.434 |
| ***ABCB1* haplotype ^a^** |  |  |  |  |  |  |
| other/other+*TTT*/other | 12 | 58 | Reference |  | Reference |  |
| *TTT/TTT* | 2 | 6 | 1.92 (0.24, 11.1) | 0.4817 | 1.56 (0.18, 10.28) | 0.6530 |
| **rs1045642** |  |  |  |  |  |  |
| *CC+CT* | 11 | 55 | Reference |  | Reference |  |
| *TT* | 3 | 9 | 2.00 (0.38, 8.95) | 0.3762 | 2.36 (0.39, 12.65) | 0.3180 |
| **rs1045642** |  |  |  |  |  |  |
| *CC* | 5 | 23 | Reference |  | Reference |  |
| *TT+CT* | 9 | 41 | 1.01 (0.30, 3.72) | 0.9843 | 0.66 (0.16, 2.70) | 0.5500 |
| **rs2032582** |  |  |  |  |  |  |
| *AA+AG+GG+AT+GT* | 11 | 54 | Reference |  | Reference |  |
| *TT* | 3 | 10 | 1.68 (0.32, 7.18) | 0.5018 | 1.66 (0.28, 8.37) | 0.5500 |
| **rs2032582** |  |  |  |  |  |  |
| *AA+AG+GG* | 7 | 27 | Reference |  | Reference |  |
| *TT+AT+GT* | 7 | 37 | 0.67 (0.20, 2.23) | 0.5045 | 0.46 (0.11, 1.73) | 0.2560 |
| **rs1128503** |  |  |  |  |  |  |
| *CC+CT* | 6 | 38 | Reference |  | Reference |  |
| *TT* | 7 | 25 | 2.07 (0.60, 7.38) | 0.2465 | 2.38 (0.62, 9.94) | 0.211 |
| **rs1128503** |  |  |  |  |  |  |
| *CC* | 2 | 8 | Reference |  | Reference |  |
| *TT+CT* | 11 | 55 | 0.75 (0.15, 5.59) | 0.7447 | 0.37 (0.04, 3.59) | 0.3550 |
| **rs1933437** |  |  |  |  |  |  |
| *CC+CT* | 4 | 31 | Reference |  | Reference |  |
| *TT* | 10 | 32 | 3.07 (0.89, 12.4) | 0.0882 | 3.55 (0.88, 18.30) | 0.0922 |
| **rs2231142** |  |  |  |  |  |  |
| *CC+AC* | 5 | 24 | Reference |  | Reference |  |
| *AA* | 9 | 38 | 1.18 (0.35, 4.34) | 0.7938 | 1.19 (0.31, 4.93) | 0.7990 |
| **rs2305948** |  |  |  |  |  |  |
| *CC* | 10 | 42 | Reference |  | Reference |  |
| *CT* | 3 | 20 | 0.56 (0.12, 2.14) | 0.4296 | 0.48 (0.08, 2.06) | 0.3520 |
| **Elevation of aspartate transaminase from a normal baseline (n=67)** | | | | | | |
| **Age** | 20 | 67 | 0.99 (0.95, 1.04) | 0.7588 |  |  |
| **Gender** |  |  |  |  |  |  |
| Male | 16 | 50 | Reference |  |  |  |
| Female | 4 | 17 | 0.65 (0.16, 2.19) | 0.5115 |  |  |
| **Baseline ECOG** |  |  |  |  |  |  |
| 0 | 9 | 23 | Reference |  |  |  |
| 1 | 7 | 33 | 0.42 (0.12, 1.36) | 0.1491 |  |  |
| 2 | 3 | 8 | 0.93 (0.16, 4.82) | 0.9350 |  |  |
| 3 | 1 | 3 | 0.78 (0.03, 9.33) | 0.8464 |  |  |
| **Starting dose** |  |  |  |  |  |  |
| <37.5mg | 0 | 1 | Reference |  |  |  |
| 37.5mg | 16 | 57 | - | 0.9920 |  |  |
| 50mg | 4 | 9 | - | 0.9916 |  |  |
| ***ABCB1* haplotype ^a^** |  |  |  |  |  |  |
| other/other | 8 | 32 | Reference |  | Reference |  |
| *TTT/TTT*+*TTT*/other | 12 | 35 | 1.57 (0.55, 4.66) | 0.4082 | 1.53 (0.49, 4.95) | 0.4680 |
| ***ABCB1* haplotype ^a^** |  |  |  |  |  |  |
| other/other+*TTT*/other | 17 | 62 | Reference |  | Reference |  |
| *TTT/TTT* | 3 | 5 | 3.97 (0.61, 32.2) | 0.1493 | 4.36 (0.58, 40.47) | 0.1540 |
| **rs1045642** |  |  |  |  |  |  |
| *CC+CT* | 16 | 59 | Reference |  | Reference |  |
| *TT* | 4 | 8 | 2.69 (0.57, 12.6) | 0.1965 | 2.41 (0.48, 11.91) | 0.2690 |
| **rs1045642** |  |  |  |  |  |  |
| *CC* | 7 | 23 | Reference |  | Reference |  |
| *TT+CT* | 13 | 44 | 0.96 (0.32, 2.99) | 0.9398 | 0.74 (0.22, 2.54) | 0.6320 |
| **rs2032582** |  |  |  |  |  |  |
| *AA+AG+GG+AT+GT* | 16 | 58 | Reference |  | Reference |  |
| *TT* | 4 | 9 | 2.10 (0.47, 8.94) | 0.3110 | 2.71 (0.54, 13.89) | 0.2170 |
| **rs2032582** |  |  |  |  |  |  |
| *AA+AG+GG* | 8 | 28 | Reference |  | Reference |  |
| *TT+AT+GT* | 12 | 39 | 1.11 (0.39, 3.31) | 0.8463 | 1.14 (0.37, 3.63) | 0.8210 |
| **rs1128503** |  |  |  |  |  |  |
| *CC+CT* | 9 | 40 | Reference |  | Reference |  |
| *TT* | 9 | 24 | 2.07 (0.68, 6.38) | 0.2002 | 2.8 (0.83, 10.03) | 0.102 |
| **rs1128503** |  |  |  |  |  |  |
| *CC* | 2 | 8 | Reference |  | Reference |  |
| *TT+CT* | 16 | 56 | 1.20 (0.25, 8.78) | 0.8337 | 1.57 (0.26, 14.43) | 0.6480 |
| **rs1933437** |  |  |  |  |  |  |
| *CC+CT* | 9 | 31 | Reference |  | Reference |  |
| *TT* | 11 | 35 | 1.12 (0.39, 3.27) | 0.8326 | 0.98 (0.32, 3.02) | 0.9740 |
| **rs2231142** |  |  |  |  |  |  |
| *CC+AC* | 8 | 27 | Reference |  | Reference |  |
| *AA* | 12 | 38 | 1.10 (0.38, 3.29) | 0.8668 | 1.02 (0.32, 3.27) | 0.9780 |
| **rs2305948** |  |  |  |  |  |  |
| *CC* | 13 | 42 | Reference |  | Reference |  |
| *CT* | 6 | 22 | 0.84 (0.25, 2.57) | 0.7597 | 0.89 (0.25, 2.91) | 0.844 |
| **Hand-foot syndrome (n=94)** | | | | | | |
| **Age** | 27 | 94 | 1.02 (0.98, 1.06) | 0.3500 |  |  |
| **Gender** |  |  |  |  |  |  |
| Male | 21 | 73 | Reference |  |  |  |
| Female | 6 | 21 | 0.99 (0.32, 2.81) | 0.9861 |  |  |
| **Baseline ECOG** |  |  |  |  |  |  |
| 0 | 7 | 27 | Reference |  |  |  |
| 1 | 18 | 49 | 1.66 (0.60, 4.92) | 0.3393 |  |  |
| 2 | 1 | 12 | 0.26 (0.01, 1.74) | 0.2340 |  |  |
| 3 | 1 | 6 | 0.57 (0.03, 4.44) | 0.6354 |  |  |
| **Starting dose** |  |  |  |  |  |  |
| <37.5mg | 0 | 5 | Reference |  |  |  |
| 37.5mg | 24 | 78 | - | 0.9924 |  |  |
| 50mg | 3 | 11 | - | 0.9925 |  |  |
| ***ABCB1* haplotype ^a^** |  |  |  |  |  |  |
| other/other | 12 | 44 | Reference |  | Reference |  |
| *TTT/TTT*+*TTT*/other | 15 | 49 | 1.18 (0.48, 2.93) | 0.7233 | 1.33 (0.51, 3.57) | 0.5670 |
| ***ABCB1* haplotype ^a^** |  |  |  |  |  |  |
| other/other+*TTT*/other | 26 | 85 | Reference |  | Reference |  |
| *TTT/TTT* | 1 | 8 | 0.32 (0.02, 1.95) | 0.3034 | 0.21 (0.01, 1.4) | 0.1686 |
| **rs1045642** |  |  |  |  |  |  |
| *CC+CT* | 23 | 81 | Reference |  | Reference |  |
| *TT* | 4 | 12 | 1.26 (0.31, 4.43) | 0.7255 | 1.21 (0.28, 4.75) | 0.7870 |
| **rs1045642** |  |  |  |  |  |  |
| *CC* | 10 | 33 | Reference |  | Reference |  |
| *TT+CT* | 17 | 60 | 0.91 (0.36, 2.36) | 0.8413 | 1.06 (0.39, 2.99) | 0.9040 |
| **rs2032582** |  |  |  |  |  |  |
| *AA+AG+GG+AT+GT* | 24 | 81 | Reference |  | Reference |  |
| *TT* | 3 | 13 | 0.71 (0.15, 2.57) | 0.6291 | 0.71 (0.14, 2.83) | 0.6400 |
| **rs2032582** |  |  |  |  |  |  |
| *AA+AG+GG* | 10 | 37 | Reference |  | Reference |  |
| *TT+AT+GT* | 17 | 57 | 1.15 (0.46, 2.95) | 0.7697 | 1.38 (0.52, 3.83) | 0.5300 |
| **rs1128503** |  |  |  |  |  |  |
| *CC+CT* | 18 | 56 | Reference |  | Reference |  |
| *TT* | 9 | 34 | 0.76 (0.29, 1.93) | 0.5697 | 0.55 (0.19, 1.5) | 0.2570 |
| **rs1128503** |  |  |  |  |  |  |
| *CC* | 1 | 8 | Reference |  | Reference |  |
| *TT+CT* | 26 | 82 | 3.25 (0.54, 62.4) | 0.2818 | 2.72 (0.38, 54.83) | 0.3830 |
| **rs1933437** |  |  |  |  |  |  |
| *CC+CT* | 15 | 47 | Reference |  | Reference |  |
| *TT* | 12 | 45 | 0.78 (0.31, 1.91) | 0.5809 | 0.76 (0.29, 2.00) | 0.5860 |
| **rs2231142** |  |  |  |  |  |  |
| *CC+AC* | 12 | 44 | Reference |  | Reference |  |
| *AA* | 14 | 48 | 1.10 (0.44, 2.76) | 0.8403 | 1.00 (0.38, 2.68) | 0.9950 |
| **rs2305948** |  |  |  |  |  |  |
| *CC* | 19 | 61 | Reference |  | Reference |  |
| *CT* | 7 | 30 | 0.67 (0.23, 1.78) | 0.4394 | 0.65 (0.21, 1.84) | 0.4260 |
| ^a^ *ABCB1 3435C/T*, *1236C/T*, *2677G/T* haplotype. | | | | | | |
