# Supplementary material for: Association of ABCB1 and FLT3 Polymorphisms with Toxicities and Survival in Asian Patients Receiving Sunitinib for Renal Cell Carcinoma
Source: PLoS One. 2015 Aug 5;10(8):e0134102. doi: 10.1371/journal.pone.0134102 (PMC4526634; doi:10.1371/journal.pone.0134102)
Supplement: S4 Table — (DOC) [file pone.0134102.s004.doc]

| S4 Table. Genotypes with non-significant associations with survival in mRCC patients receiving sunitinib as first-line treatment (n=81) | | | | | | | |
| --- | --- | --- | --- | --- | --- | --- | --- |
| Factor | | No. | Median  (months) | Univariate | | Multivariate^a^ | |
|  |  |  |  | HR (95% CI) | *P* | HR (95% CI) | *P* |
| **Progression-free survival** | | | | | | | |
| *VEGFR2 1191 C/T* | *CC*  *CT* | 55  24 | 8.4  5.0 | 1  1.5 (0.8, 2.9) | 0.18 | 1  1.3 (0.7, 2.6) | 0.41 |
| *FLT3*  *738 T/C* | *CC*+*CT*  *TT* | 40  40 | 8.1  8.4 | 1  0.9 (0.5, 1.5) | 0.59 | 1  1.0 (0.6, 1.7) | 0.92 |
| *ABCG2 421 C/A* | *CC*+*AC*  *AA* | 39  40 | 8.1  7.8 | 1  1.2 (0.7, 2.1) | 0.49 | 1  1.3 (0.7, 2.4) | 0.38 |
| **Overall survival** | | | | | | | |
| *VEGFR2 1191 C/T* | *CC*  *CT* | 55  24 | 19.9  10.1 | 1  1.3 (0.7, 2.3) | 0.45 | 1  1.0 (0.6, 1.9) | 0.96 |
| *FLT3*  *738 T/C* | *CC*+*CT*  *TT* | 40  40 | 18.6  19.5 | 1  0.8 (0.5, 1.4) | 0.45 | 1  0.9 (0.5, 1.6) | 0.72 |
| *ABCG2 421 C/A* | *CC*+*AC*  *AA* | 39  40 | 16.3  20.7 | 1  1.0 (0.6, 1.6) | 0.86 | 1  1.1 (0.6, 1.9) | 0.82 |
| Abbreviations: HR, hazard ratio; CI, confidence interval.  ^a^ including starting dose as covariate. | | | | | | | |
