# Supplementary material for: Association of ABCB1 and FLT3 Polymorphisms with Toxicities and Survival in Asian Patients Receiving Sunitinib for Renal Cell Carcinoma
Source: PLoS One. 2015 Aug 5;10(8):e0134102. doi: 10.1371/journal.pone.0134102 (PMC4526634; doi:10.1371/journal.pone.0134102)
Supplement: S5 Table — (DOC) [file pone.0134102.s005.doc]

| S5 Table. Sample size estimation for the validation of previous associations. Genotype frequencies in the Chinese population were collected from National Center for Biotechnology Information, and the haplotype frequency for *ABCB1 3435*, *1236*, *2677 TTT* was obtained from Tang *et al.*. Our study was designed to enroll patients who started sunitinib therapy during an 84-month accrual period from late 2006 to early 2014, and to continue follow-up for 3 months after end of patient recruitment. Calculation was conducted using the software Power and Sample Size Calculation . | | | |
| --- | --- | --- | --- |
| Type I error (α) |  | Power (1-β) |  |
|  | **0.90** | **0.80** | **0.70** |
|  | **Poorer PFS with the *ABCB1 1236 TT* genotype** | | |
| **0.05** | 88 | 66 | 52 |
| **0.01** | 124 | 96 | 80 |
|  | **Poorer OS with the *ABCB1 1236 TT* genotype** | | |
| **0.05** | 102 | 76 | 60 |
| **0.01** | 144 | 114 | 94 |
|  | **Leucopenia increased with *FLT3 738 TT* genotype** | | |
| **0.05** | 279 | 213 | 172 |
| **0.01** | 385 | 308 | 258 |
|  | **Hand-foot syndrome with the *ABCB1 3435, 1236, 2677 TTT* haplotype** | | |
| **0.05** | 286 | 223 | 181 |
| **0.01** | 399 | 321 | 272 |

**References for this table**

(1) Tang, K. *et al.* Distinct haplotype profiles and strong linkage disequilibrium at the MDR1 multidrug transporter gene locus in three ethnic Asian populations. *Pharmacogenetics* **12**, 437-50 (2002).

(2) Dupont, W.D. & Plummer, W.D., Jr. Power and sample size calculations. A review and computer program. *Controlled clinical trials* **11**, 116-28 (1990).

(3) Beuselinck, B. *et al.* Single-nucleotide polymorphisms associated with outcome in metastatic renal cell carcinoma treated with sunitinib. *British journal of cancer* **108**, 887-900 (2013).

(4) van Erp, N.P. *et al.* Pharmacogenetic pathway analysis for determination of sunitinib-induced toxicity. *J Clin Oncol* **27**, 4406-12 (2009).
