# Supplementary material for: Association of ABCB1 and FLT3 Polymorphisms with Toxicities and Survival in Asian Patients Receiving Sunitinib for Renal Cell Carcinoma
Source: PLoS One. 2015 Aug 5;10(8):e0134102. doi: 10.1371/journal.pone.0134102 (PMC4526634; doi:10.1371/journal.pone.0134102)
Supplement: S6 Table — (DOC) [file pone.0134102.s006.doc]

| S6 Table. Primers for Genotyping | | | | |
| --- | --- | --- | --- | --- |
| Gene | Polymorphism | rs Number | Forward Primer | Reverse Primer |
| *VEGFR2* | 1191 C/T | rs2305948 | 5’TCTTGGTCATCAGCCCACTG3’ | 5’AAACCCAGTCTGGGAGTGAG3’ |
| *FLT3* | 738 T/C | rs1933437 | 5’GCAGCTGTAAAGAAGAAAGTCCAG3’ | 5’TGCATTCCCTGCCCAGTT3’ |
| *ABCB1* | 1236 T/C | rs1128503 | 5’TGTCTGTGAATTGCCTTGAA3’ | 5’GGTCATAGAGCCTCTGCATC3’ |
| *ABCB1* | 2677 G/TA | rs2032582 | 5’GTACCCATCATTGCAATAGCA3’ | 5’TTTAGTTTGACTCACCTTCCCAG3’ |
| *ABCB1* | 3435 C/T | rs1045642 | 5’GAGCCCATCCTGTTTGACTGC3’ | 5’CATTAGGCAGTGACTCGATGAAGGC3’ |
| *ABCG2* | 421 C/A | rs2231142 | 5’GGATGATGTTGTGATGGGCACTC3’ | 5’CCAGACCTAACTCTTGAATGACCCTG3’ |
| *BIM* | *BIM* i2del^a^ | - | 5’CTTAGCGTAATG TCGTCAGGG3’  5’AGGCTTCAGTGA GGTAAATCAC3’ | 5’GCTCCTCTGTGAGGC CAGCCTG3’ |
| ^a^ a 2,903-bp deletion polymorphism in intron 2 of *BIM* previously associated with resistance to tyrosine kinase inhibitors . | | | | |

**References for this table**

(1) Kim, J.J. *et al.* Association of VEGF and VEGFR2 single nucleotide polymorphisms with hypertension and clinical outcome in metastatic clear cell renal cell carcinoma patients treated with sunitinib. *Cancer* **118**, 1946-54 (2012).

(2) Onizuka, M. *et al.* Cytochrome P450 genetic polymorphisms influence the serum concentration of calcineurin inhibitors in allogeneic hematopoietic SCT recipients. *Bone marrow transplantation* **46**, 1113-7 (2011).

(3) Jeannesson, E. *et al.* Determination of ABCB1 polymorphisms and haplotypes frequencies in a French population. *Fundamental & clinical pharmacology* **21**, 411-8 (2007).

(4) Ng, K.P. *et al.* A common BIM deletion polymorphism mediates intrinsic resistance and inferior responses to tyrosine kinase inhibitors in cancer. *Nature medicine* **18**, 521-8 (2012).
